# **Increased Life Span due to Calorie** **Restriction in Respiratory-Deficient Yeast**

**DOI:** 10.1371/journal.pgen.0020033

**Published:** 2006-03-31

**Authors:** Su-Ju Lin, Leonard Guarente

The Kaeberlein et al. paper in the November 2005 issue of *PLoS Genetics* [1] claimed that calorie restriction (CR) in yeast does not require respiration to extend life span of mother cells. This claim challenges our earlier finding that deleting CYT1 (encoding cytochrome c1) prevented CR-associated longevity [2].

However, there is a fundamental difference in the two experiments: these authors typically use 0.05% glucose as their CR media instead of the 0.5% glucose in our experiments (compared with 2% glucose in controls). At their very low glucose levels, yeast cells are slow growing and show significant metabolic changes [3].

Moreover, the key for interpreting the effect of the *cyt1* deletion on CR is to examine the effect of the deletion on life span at a given reduced glucose concentration (compared with the usual 2%) and comparing this with the degree of life extension in the wild-type parental strain at that same reduced glucose concentration. The single experiment the authors present using our strain (PSY316) and our glucose concentration (0.5%) actually showed very little extension (at the margin of significance). Unfortunately, even this small effect is misleading because the experiment omits the wild-type control. In fact, an earlier study by Kaeberlein showed robust extension of life span at 0.5% glucose in the wild-type parental strain PSY316 [4]. The omission of this information from the experiment in Figure 3A obscures the key fact that deleting CYT1 evidently did prevent most of the extension in life span by 0.5% glucose in their hands, which would agree with our previous findings.

The authors did find a much more robust extension at 0.05% glucose in the *cyt1* deletion in Figure 3A, a condition we did not examine [2]. The authors explain away the weak effect they see at 0.5% glucose in the *cyt1* mutant compared with the much larger effect at 0.05% glucose by saying a “non-optimal level of CR may have precluded detection of lifespan extension by CR in the *cyt1* deletion mutants in the prior study” [1]. But, as mentioned above, an earlier study by Kaeberlein showed a robust life extension in the wild-type parent PSY316 at 0.5% glucose, which was as great as that observed at 0.05% [4]. Indeed, 0.5% glucose was chosen as our standard in this strain because life span was maximal and the growth rate was reasonably rapid.

Therefore, the claims of Kaeberlein et al. that respiration and, by implication, SIR2-related genes are not required for CR-induced longevity are highly misleading. We previously showed the requirement for SIR2 in PSY316 [2], and, moreover, Lamming et al. [5] recently showed the requirement for SIR2 and related paralogs in the strain commonly used by Kaeberlein et al. (using 0.5% glucose). We think it is likely that different pathways are engaged at 0.05% compared with 0.5% glucose. Thus, their claim that respiration is not required for longevity may apply to their experimental conditions, but not to ours. In summary, we think it is likely that differences in pathways identified by Kaeberlein et al. simply reflect their different experimental protocols and do not negate our earlier findings and interpretations. Futhermore, the fact that they changed the conditions we employed as CR and omitted relevant data have created confusion.

## References

[pgen-0020033-b001] Kaeberlein M, Hu D, Kerr EO, Tsuchiya M, Westman EA (2005). Increased life span due to calorie restriction in respiratory-deficient yeast. PLoS Genet.

[pgen-0020033-b002] Lin SJ, Kaeberlein M, Andalis AA, Sturtz LA, Defossez PA (2002). Calorie restriction extends Saccharomyces cerevisiae life span by increasing respiration. Nature.

[pgen-0020033-b003] Brauer MJ, Saldanha AJ, Dolinski K, Botstein D (2005). Homeostatic adjustment and metabolic remodeling in glucose-limited yeast cultures. Mol Biol Cell.

[pgen-0020033-b004] Kaeberlein M, Andalis AA, Fink GR, Guarente L (2002). High osmolarity extends life span in Saccharomyces cerevisiae by a mechanism related to calorie restriction. Mol Cell Biol.

[pgen-0020033-b005] Lamming DW, Latorre-Esteves M, Medvedik O, Wong SN, Tsang FA (2005). HST2 mediates SIR2-independent life-span extension by calorie restriction. Science.

